# Development of a downstream process for the production of an inactivated whole hepatitis C virus vaccine

**DOI:** 10.1038/s41598-020-72328-5

**Published:** 2020-10-01

**Authors:** Keven Lothert, Anna F. Offersgaard, Anne F. Pihl, Christian K. Mathiesen, Tanja B. Jensen, Garazi Peña Alzua, Ulrik Fahnøe, Jens Bukh, Judith M. Gottwein, Michael W. Wolff

**Affiliations:** 1 Institute of Bioprocess Engineering and Pharmaceutical Technology, Department of Life Science Engineering, University of Applied Sciences Mittelhessen (THM), Giessen, Germany; 2 Copenhagen Hepatitis C Program (CO-HEP), Department of Infectious Diseases, Hvidovre Hospital and Department of Immunology and Microbiology, Faculty of Health and Medical Sciences, University of Copenhagen, Copenhagen, Denmark; 3 Fraunhofer Institute for Molecular Biology and Applied Ecology (IME), Giessen, Germany

**Keywords:** Infectious diseases, Biotechnology

## Abstract

There is a large unmet need for a prophylactic hepatitis C virus (HCV) vaccine to control the ongoing epidemic with this deadly pathogen. Many antiviral vaccines employ whole viruses as antigens. For HCV, this approach became feasible following the development of infectious cell culture systems for virus production. However, the lack of efficient downstream processes (DSP) for HCV purification poses a roadblock for the development of a whole virus vaccine. Using cell culture-derived genotype 1a HCV we developed a scalable and efficient DSP train, employing commonly used clarification and ultrafiltration techniques, followed by two membrane-based chromatography steps. For virus capture, steric exclusion chromatography using cellulose membranes was established, resulting in a virtually complete virus recovery with > 99% protein and 84% DNA depletion. Virus polishing was achieved by sulphated cellulose membrane adsorbers with ~ 50% virus recovery and > 99% protein and 90% DNA depletion. Additional nuclease digestion resulted in 99% overall DNA depletion with final DNA concentrations of 2 ng/mL. Process results were comparable for cell culture-derived HCV of another major genotype (5a). This study provides proof-of-concept for establishment of an efficient and economically attractive DSP with potential application for production of an inactivated whole virus vaccine against HCV for human use.

## Introduction

The hepatitis C virus (HCV) is a small enveloped virus, 30–80 nm in diameter^1,2^, with a single positive stranded RNA genome, belonging to the *Flaviviridae* family^3,4^. The RNA genome encodes 3 structural proteins, the capsid protein Core, and the envelope glycoproteins E1 and E2, which are incorporated into the viral particle, as well as 7 nonstructural proteins (p7, NS2, NS3, NS4A, NS4B, NS5A, and NS5B). There are 8 different major genotypes, differing in ~ 30% of their nucleotide and amino acid sequence, with genotype 1 being most frequent worldwide^5,6^.

Each year, there are at least 2 million new HCV infections, of which ~ 80% result in chronic infections. There are at least 71 million chronically infected individuals worldwide with an increased risk of liver cirrhosis and hepatocellular carcinoma, resulting in ~ 400,000 deaths per year^7–9^.

Only a minor fraction of HCV-infected individuals are treated with recently licensed efficient direct-acting antivirals (DAA). The main reasons for this are that most individuals are not aware of their infection status, as the infection is typically asymptomatic until a severe and often irreversible liver disease has developed, and because of the lack of screening programs and the high cost of DAA. Furthermore, resistance to DAA is increasing and might compromise future treatment efficacy^10,11^. Thus, a vaccine is urgently needed to control HCV on a global scale^12–14^.

Many antiviral vaccines are based on viral particles as vaccine antigens^15,16^ and protect by their induction of neutralizing antibodies. The proof-of-concept for the immunogenicity of cell culture-derived inactivated HCV has been obtained in animal models^17–19^. However, in these studies, ultracentrifugation-based downstream processes (DSP) were employed for virus concentration and purification. This approach is in general characterized by a relatively low recovery, a limited scalability, and a limited impurity depletion. Thus, as for most other vaccines, the development of an efficient DSP, compatible with industrial requirements, is a major bottleneck for the manufacturing of a whole virus HCV vaccine for human use^20–22^.

Here, we evaluated commonly used clarification and ultrafiltration in combination with two membrane-based chromatography technologies, (1) steric exclusion chromatography (SXC), and (2) chromatography based on sulphated cellulose membrane adsorbers (SCMA), for the development of a cost-efficient and scalable DSP, compatible with good manufacturing practices (GMP). SXC was initially described for the purification of bacteriophages and large proteins, using hydrophilic monoliths and starch-coated magnetic nanoparticles^23–25^. With the application of unmodified cellulose membranes, SXC also proved to be a valuable tool for the purification of different viruses with recoveries of 99% (Influenza A^26^) and 91% (Baculovirus^27^). The method is based on the steric exclusion of particles in a solution of an inert polymer, e.g. polyethylene glycol (PEG). This exclusion leads to the formation of polymer-rich and polymer-deficient zones, resulting in thermodynamic instability, which is resolved by an association of the excluded particles with each other, and with the hydrophilic stationary phase, thus, retaining target molecules from the mobile phase. A careful adjustment of the PEG concentration and the molecular weight, with regard to the size of the product and expected process impurities, allows a selective product retention. Retained particles are eluted by the removal of the inert polymer from the mobile phase^26,27^. As the method highly depends on the size of the product, it is important to mention that HCV is thought to be smaller than the viruses used in previous publications on membrane-based SXC. Additionally, it has previously been described, that the retention works best near the isoelectric point (pI) of the target molecule^28^.

SCMA was applied for subsequent polishing, employing a pseudo affinity-based orthogonal technique. The method has been described for the purification of the Influenza A virus and the Modified Vaccinia Ankara virus^29–33^. It utilizes the heparin-mimicking effect of sulphated cellulose and should, thus, be widely applicable to viruses with an affinity to heparin. As HCV was successfully purified from infected plasma using a heparin chromatography resin, such an affinity could be expected^34^.

The aim of this study was to provide proof-of-concept for the development of an efficient, scalable, and GMP-compatible DSP for the purification of cell culture-derived HCV to eventually facilitate an industrial production of a human HCV vaccine. In summary, the evaluated process unit operations included clarification, ultrafiltration, nuclease treatment as well as SXC and SCMA as chromatographic capture and polishing steps.

## Results

### Production of high-titre HCV genotype 1a virus stock for DSP development

We first focussed on processing HCV genotype 1 being the most prevalent HCV genotype worldwide. However, to facilitate DSP development, a high-titre variant efficiently producing infectious viruses in cell culture was required. Thus, the previously reported recombinant genotype 1a virus TNcc^35^ was serially passaged in naïve human hepatoma cell line 7.5 (Huh7.5) cells for a further adaptation to the cell culture, until HCV infectivity titres of ~ 6 log_10_ focus forming units (FFU)/mL, were observed for several passages. A passage 19 stock was prepared, serving as the seed for the genotype 1a passage 20 virus production in triple layer culture flasks, which was then used in the DSP development. Next generation sequencing (NGS) revealed that, in addition to the eight cell culture adaptive substitutions in the original 1a virus recombinant, passage 20 viruses had acquired 3 substitutions present in > 50% of the virus population, G1909A in NS4B as well as N2651H and H2986R in NS5B (Supplementary Table S1). Overall, the largest heterogeneity was observed in the nonstructural proteins.

### HCV clarification and ultrafiltration

A two-step filtration, with cut-offs of 5 µm and 0.65 µm, was selected for the clarification of genotype 1a HCV. Subsequently, the clarified material was concentrated in two sequential ultrafiltration steps with a cut-off of 500 kDa from a starting volume of 10.5 L to volumes of 600 mL and 42 mL, respectively. We observed a virtually complete virus recovery for the clarification and the first ultrafiltration step, whereas a 45% recovery was observed for the second ultrafiltration step. For inactivation, the resulting material was treated by UV irradiation, and naïve cell cultures were inoculated and followed for three weeks by regular immunostainings for the HCV NS5A antigen to confirm inactivation.

### HCV capture by SXC

**Figure 1:**
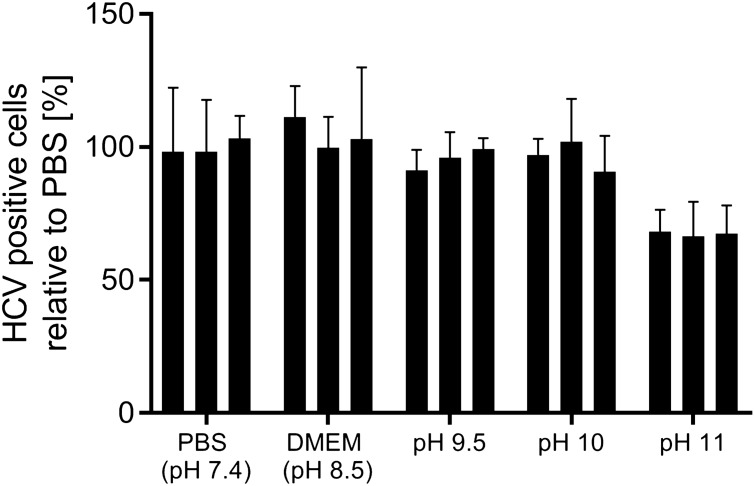
HCV stability at alkaline pH. HCV was incubated at room temperature for 90 min in PBS (pH 7.4), DMEM standard cell culture medium (pH 8.5) and phosphate buffers for alkaline conditions (pH 9.5, 10 and 11). Subsequently, solutions were neutralized with DMEM containing 20 mM HEPES and used to infect cells. The number of infected cells after 48 h of incubation was evaluated relative to the mean of the number of infected cells resulting from infection with virus incubated in PBS. Data from 3 biological replicates are shown as separate bars. Error bars are standard deviations (SD) representing 3 technical replicates.

**Figure 2:**
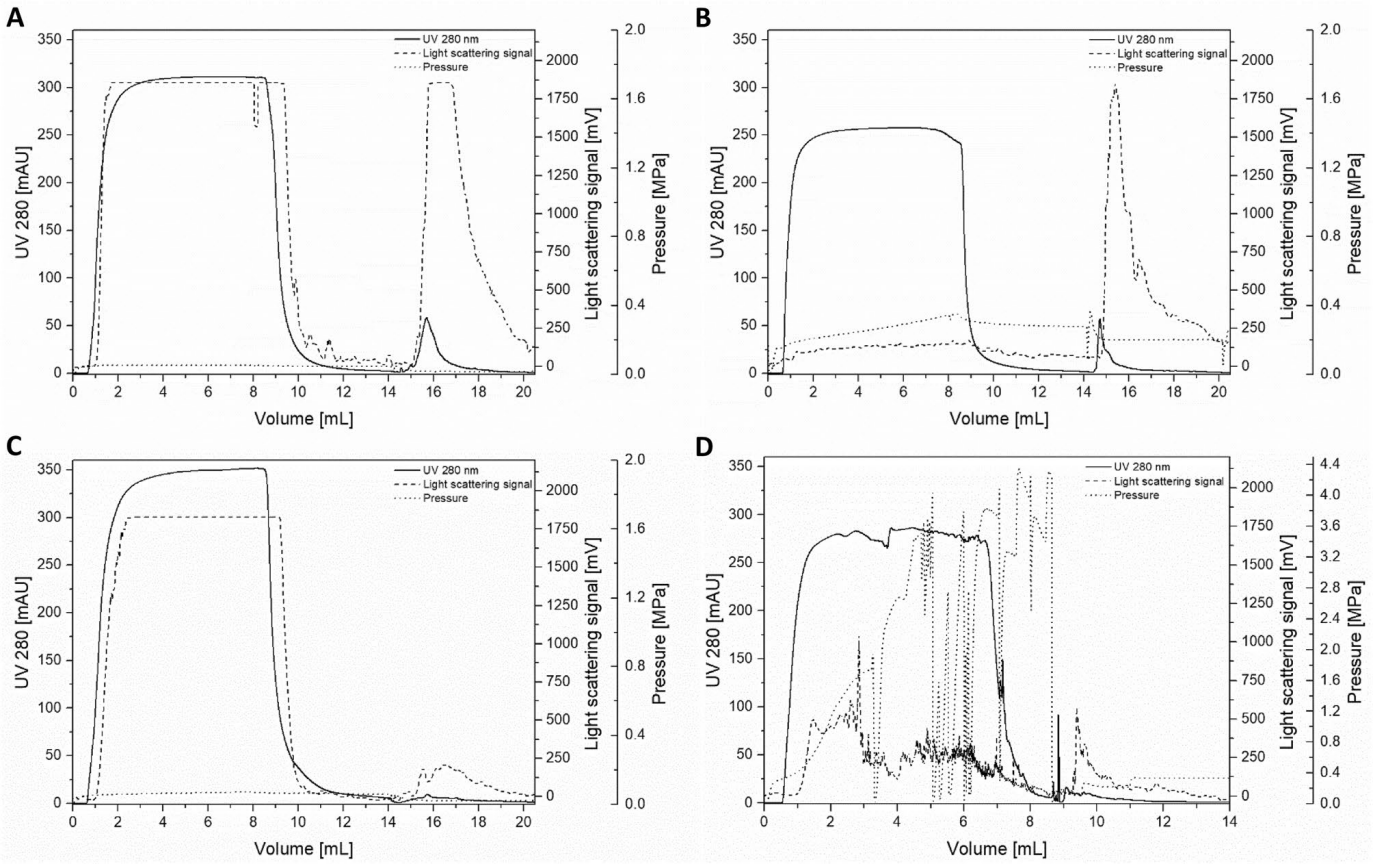
Influence of pH on SXC chromatography. For binding and washing of genotype 1a HCV 20 mM Tris with 180 mM NaCl and 8% PEG 6,000 were used at (**A**) pH 8, (**B**) pH 9 and (**C**) pH 10; loading: 0–9 mL, washing: 9–15 mL, elution (without PEG using 180 mM NaCl): 15–21 mL. (**D**) at pH 11 the flow rate was reduced at about 4 mL as the pressure already increased above 2.5 MPa. Here washing was already initiated after 6 mL.

**Figure 3:**
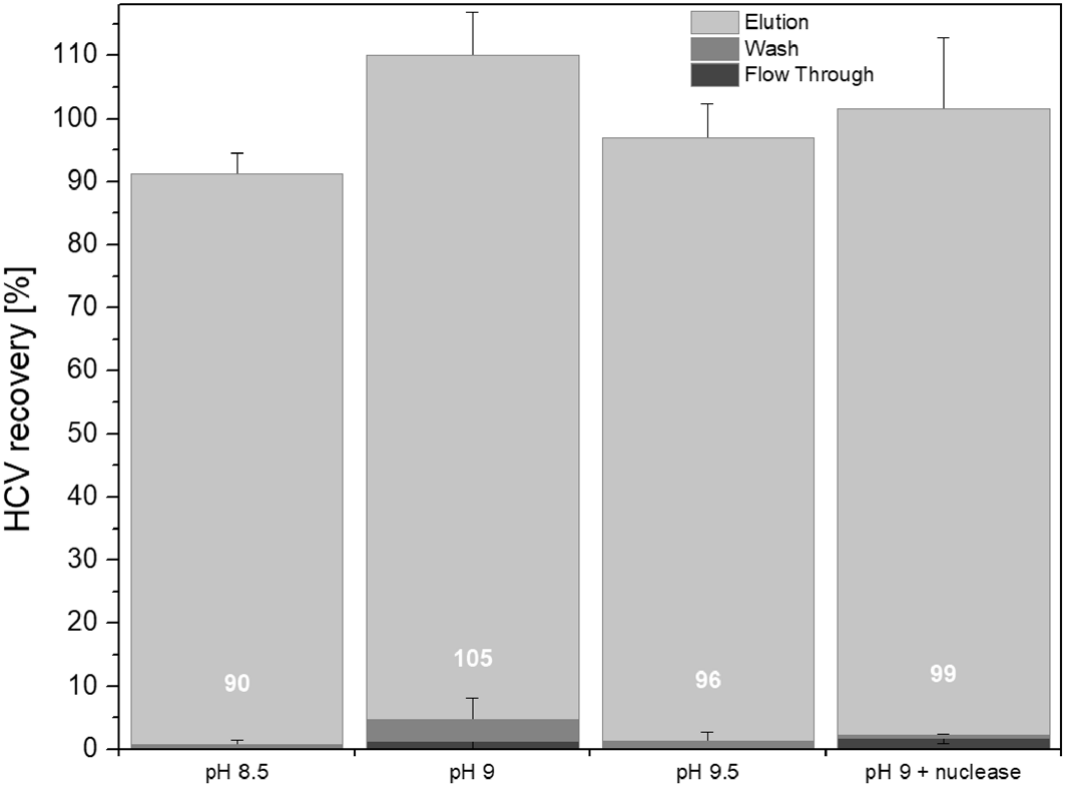
SXC HCV recoveries for different process conditions. Recovery was calculated by relating amounts of genotype 1a HCV RNA in flow through, wash and elution fractions to the total RNA amount in the feed prior to SXC. Variations included changes in the process pH and additional nuclease digestion prior to SXC. Values are means of technical triplicates with error bars reflecting SD.

When in initial SXC experiments published standard conditions with 8% PEG and physiological pH values were used^26^, the majority of the viruses were found in the flow-through fraction. An increase in the PEG concentration to up to 12% did not result in improvements. Based on prior publications, unpublished experiments and theoretical sequence-based calculations provided on viprbrc.org, we hypothesized that the pI of HCV might be alkaline^36^. Thus, prior to testing alkaline SXC conditions, we investigated HCV stability at different pH values. HCV was incubated in phosphate-buffered saline (PBS, pH 7.4), Dulbecco’s Modified Eagle Medium (DMEM, standard cell culture medium, pH 8.5), and phosphate buffers for final pH values of ~ 9.5, 10 and 11, prior to inoculation of naïve Huh7.5 cells for the determination of HCV infectivity. Of note, the tested conditions did not result in an impairment of cell viability. HCV was stable when subjected to pH values of up to 10 for 90 min (Fig. 1), which equals the approximate duration of the SXC. Testing alkaline SXC conditions revealed a large virus breakthrough at pH 8 and 10 during loading (Fig. 2A,C) based on light-scattering detection. At pH 11 a strongly increasing back pressure was observed with increasing loading volume during sample application and wash (Fig. 2D). This resulted in a reduced virus breakthrough, a decreased possible loading volume, and nearly no virus recovery in any of the fractions. In contrast, at pH 9, the virus breakthrough was minimized (Fig. 2B). In an additional experiment, the qualitative data based on the light-scattering signal, was verified by quantitative polymerase chain reaction (qPCR) analytics of the recovered viral RNA. Here, at pH 8.5, 9, and 9.5, a virtually full virus retention and recovery in the elution fraction could be achieved, with a product yield in the range of 90% to 105% (Fig. 3, Table 1). An additional nuclease treatment did not affect the SXC and resulted in similar recoveries of 99% (Fig. 3, Table 1) with minor amounts of virus found in the flow-through (2%) and wash (< 1%) fractions.

**Table 1:** Overview on viral recovery and impurity depletion for the two chromatography-based process steps and cell culture-derived genotype 1a and 5a HCV.

HCV genotype	Virus in product [IU/mL]	Virus recovery [%]	Protein in product [µg/mL]	Protein depletion [%]	DNA in product [ng/mL]	DNA depletion [%]	DNA per 1.0E+08 IU/mL [ng]
**SXC capture**							
1a (without nuclease)	2.7E+08	105 ± 7	5 ± 2	> 99	107 ± 42	84 ± 3	~ 39
1a	9.3E+07	99 ± 11	4 ± 1	> 99	9 ± 1	94 ± 2	~ 10
5a	8.1E+07	97 ± 3	15 ± 6	97 ± 2	12 ± 2	86 ± 1	~ 15
**SCMA polishing**							
1a (without nuclease)	1.7E+08	63 ± 16	< 0.5	> 99	57 ± 17	90 ± 6	~ 33
1a	3.5E+07	50 ± 16	0.9 ± 0.5	> 99	2 ± 0.5	99 ± 0.5	~ 5
5a	3.2E+07	49 ± 5	1.5 ± 0.4	> 99	3 ± 1	98 ± 1	~ 9

Shown are the values for SXC capture at pH 9 and the SCMA polishing using a TRIS buffer at pH 7.4. Recoveries are step recoveries comparing feed and product fractions of the respective step, and depletions are overall values, related to the initial feed concentrations before nuclease treatment and SXC. For a better overview, normalized DNA contents are given for each step, calculated for virus titres of 1.0E+08. While stated values for protein and DNA concentrations are rounded, values for % protein and DNA depletion as well as DNA per 1.0E+08 IU/ml were calculated using non-rounded values.

n = 3 for all steps.

The dynamic binding capacity (DBC) of the membranes was determined using 3.9E+08 international units (IU)/cm^2^ until a pressure limitation occurred and was approximately 2.1E+08 IU/cm^2^ until a 10% breakthrough was observed (DBC_10%_). However, due to an excessive pressure increase, it was not possible to load the virus until a 100% breakthrough occurred, thus DBC_100%_ could not be determined.

**Figure 4:**
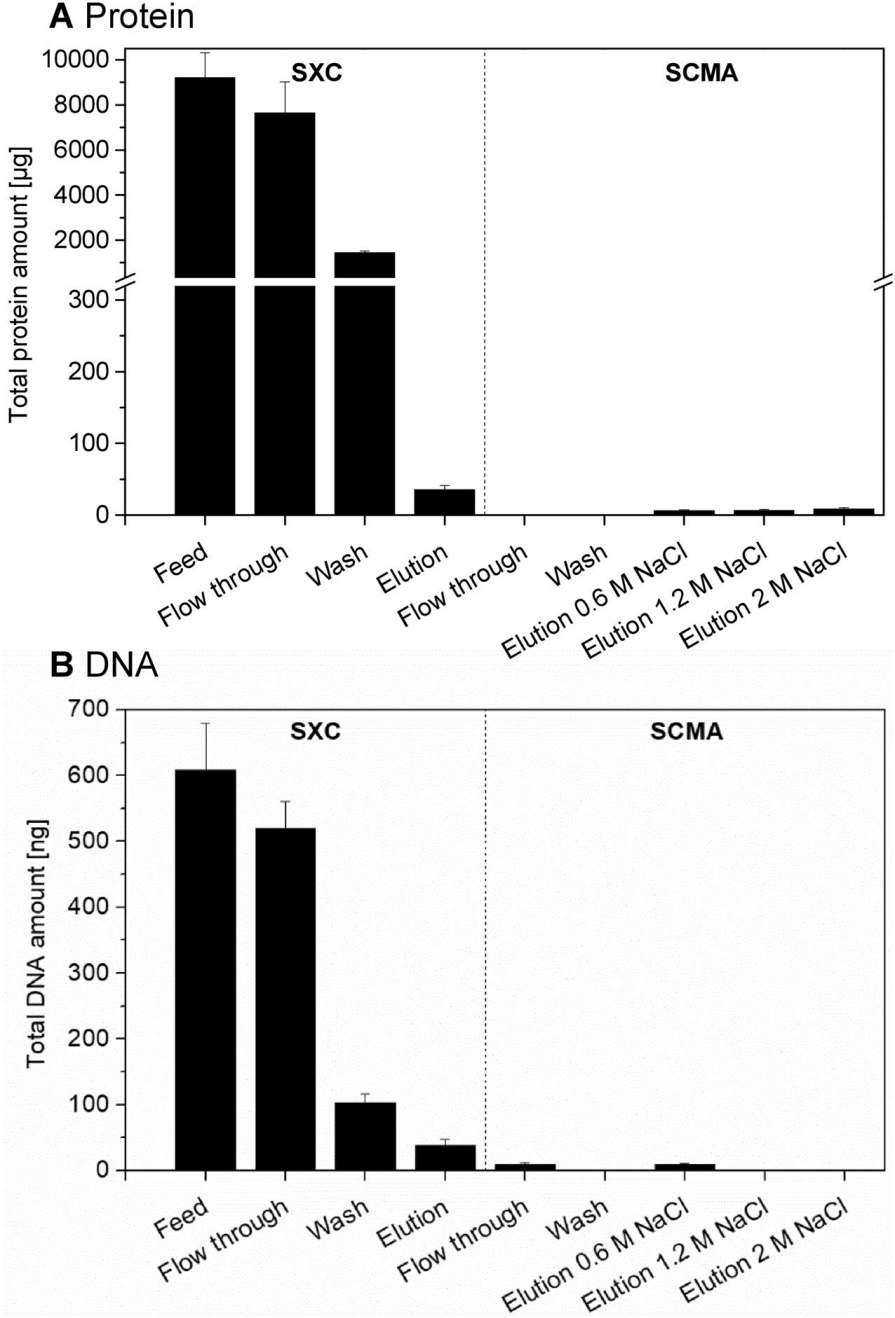
Impurity removal during SXC and SCMA with preceding nuclease treatment. Shown is the total amount of (**A**) protein and (**B**) DNA in individual fractions resulting from SXC (pH 9) of nuclease treated 1a HCV (material shown in Fig. 3) and consecutive SCMA. In the cases where no bars are visible, protein and DNA amounts were below the limit of detection of the assays (25 pg/mL for the DNA and 0.5 µg/mL for the protein assay).

The impurity removal did not depend on the pH value. For the runs without a preceding nuclease treatment as shown in Fig. 3 (pH 8.5, 9 and 9.5), the protein depletion was above 99% and the DNA depletion was 84% (data for pH 9 shown in Table 1, other data not shown). The additional nuclease digestion, followed by SXC at pH 9, did not affect the protein depletion, which was above 99% resulting in a protein concentration of 4 µg/mL (Fig. 4A, Table 1), but resulted in an increased DNA depletion of 94%, and DNA concentrations of 9 ng/mL at viral RNA titres of 9.3E+07 IU/mL after SXC (Fig. 4B, Table 1).

### HCV polishing by SCMA chromatography

**Figure 5:**
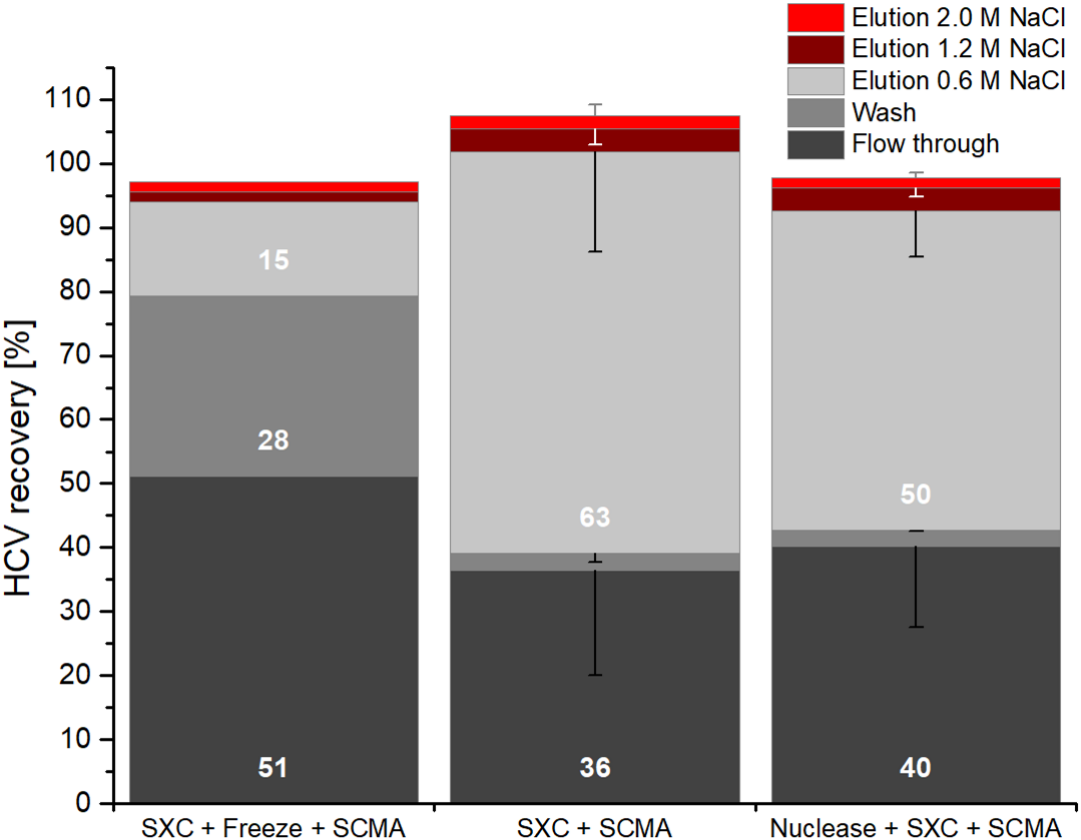
SCMA HCV recoveries for different process conditions. Recovery was calculated by relating amounts of genotype 1a HCV RNA in flow through, wash and elutions at 0.6, 1.2, and 2.0 M NaCl to the total RNA amount prior to SCMA. All preceding SXC runs were performed at pH 9 (eluate fractions of Fig. 3), without nuclease treatment prior to SXC (left and middle bar), including intermediate freezing (left bar) and with nuclease treatment preceding SXC (right bar). Bar captions state the order, in which steps were performed. Error values are means of technical triplicates with error bars reflecting SD.

The SXC elutions, resulting from the SXC experiments done at pH 9 without and with preceding nuclease treatment (Fig. 3), were further processed using SCMA. Following SXC without a preceding nuclease treatment, the HCV recovery was 63% in the 0.6 M NaCl elution fraction, when the SXC elution was directly processed without an additional freeze–thaw cycle (Fig. 5). A storage at − 80 °C in between the SXC and SCMA led to a reduction of retained and eluted viruses to 15%, with the majority of viruses found in the flow-through and wash fractions. When testing the implementation of a nuclease treatment prior to SXC, a virus recovery of 50% in the 0.6 M NaCl elution fraction was observed. In this experiment, minor amounts of virus eluted at higher salt concentrations of 1.2 and 2 M NaCl (Fig. 5), whereas 42% of the loaded virus was found in the flow-through and wash fractions.

Due to a breakthrough of about 40–50% of the virus, DBC_10%_ and DBC_100%_ could not be reached. However, the sample application was not limited by binding capacity but by pressure, as the pressure after an application of ~ 5.9E+09 IU/cm^2^ exceeded the limits.

Considering the removal of impurities, no proteins could be detected in the SCMA flow-through and wash fractions, whereas less than 1 µg/mL was found in the SCMA elution fraction obtained following SXC at pH 9 without or with preceding nuclease treatment, indicating a virtually complete protein depletion (Fig. 4A for nuclease treatment + SXC (pH 9) + SCMA; Table 1 for both datasets). The overall DNA depletion was 90% following SXC (pH 9) and SCMA without a preceding nuclease treatment compared to the initial feed concentration before SXC (Table 1). The introduction of a nuclease digestion followed by SXC (pH 9) and SCMA resulted in an increased DNA depletion of above 99% compared to the initial feed concentration before nuclease treatment and SXC, leading to DNA concentrations of about 2 ng/mL at viral RNA titres of 3.5E+07 IU/mL (Fig. 4B, Table 1).

### The developed DSP was equally efficient for different HCV genotypes

In order to investigate the applicability of the developed DSP for different HCV isolates, we applied this strategy to a high-titre cell culture-derived genotype 5a virus^37,38^, which was selected due to its efficient growth characteristics in cell culture. Compared to the genotype 1a virus, the genotype 5a structural proteins differ by ~ 20%, while the envelope proteins differ by ~ 26% on the amino acid level.

The 5a virus was produced in cell factories; NGS showed that, in comparison to the published sequence, no additional substitutions were present in > 2% of the viral population. Clarification and ultrafiltration were carried out as for the 1a virus, with a volume reduction from 20.4 L to 420 mL and 63 mL in the first and second ultrafiltration, respectively. During clarification and the first ultrafiltration, we observed a virtually complete virus recovery, whereas a recovery of 87% was observed for the second ultrafiltration step. The resulting 5a material was UV- irradiated and the inactivation was confirmed as described for the 1a material.

**Figure 6:**
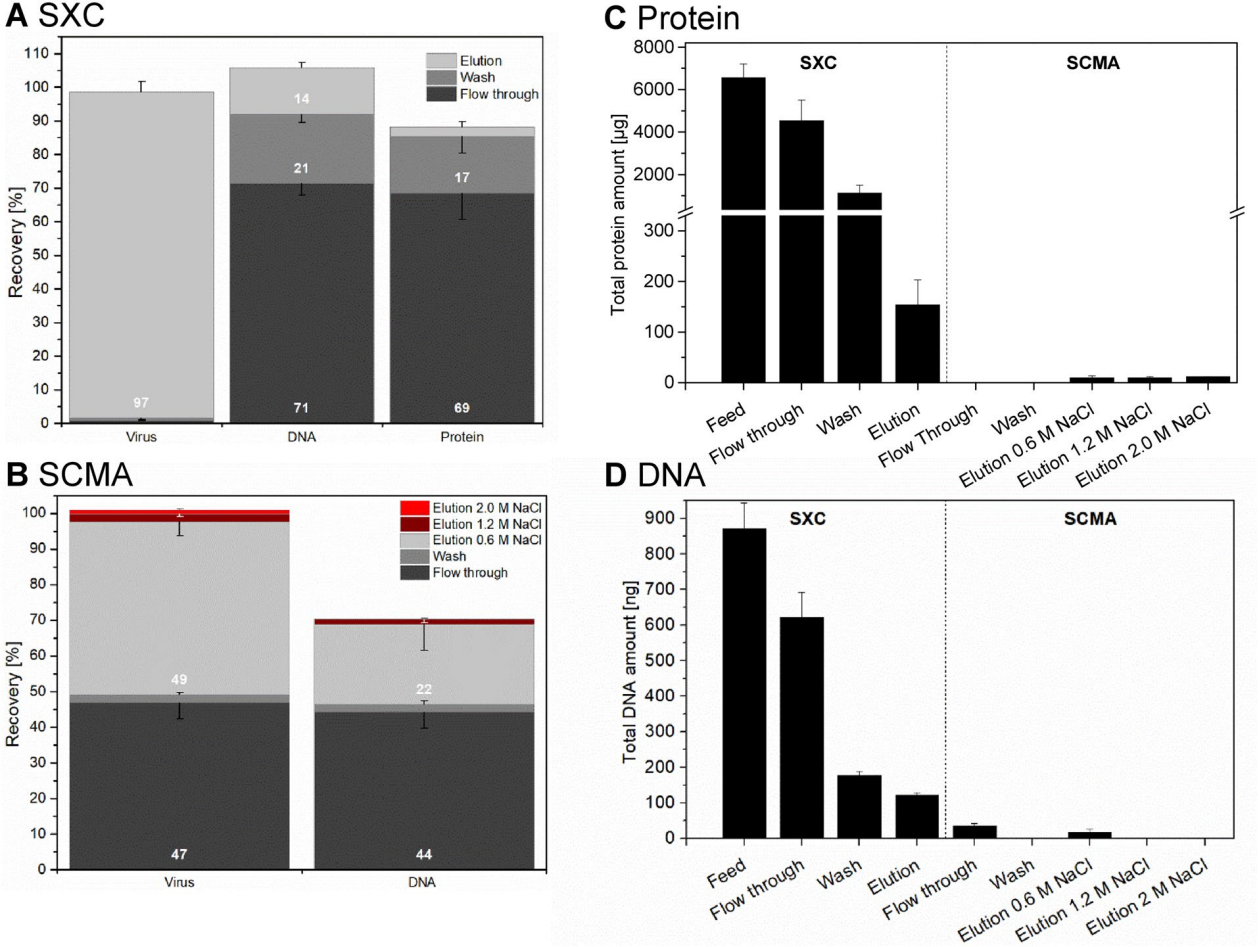
Process performance for a different HCV genotype. Shown is (**A**) the recovery of virus, DNA and protein during SXC and (**B**) the recovery of virus and DNA in the different SCMA fractions. Furthermore, (**C**) the total protein amounts and (**D**) the total DNA amounts throughout the process are depicted. All recovery values are step-recoveries, correlated to the quantities in the loading sample of the respective step. For the SCMA, no protein and DNA recoveries and amounts are depicted in case the concentration was below the limit of detection of the assay (0.5 µg/mL and 25 pg/mL for protein and DNA, respectively). Error bars indicate technical triplicates with error bars reflecting SD.

With a preceding nuclease digestion, the SXC virus recovery was 97% (Fig. 6A, Table 1). During the SXC, the DBC_10%_ was determined with 9.8E+07 IU/cm^2^ and a sample application was pressure-limited at about 2.7E+08 IU/cm^2^. SCMA was carried out directly after SXC and resulted in a virus recovery of 49% in the 0.6 M NaCl elution fraction, whereas 47% of the applied virus was lost in the flow-through (Fig. 6B, Table 1). As for the 1a virus, DBC_10%_ or DBC_100%_ could not be determined during SCMA. The whole process led to a virtually complete protein removal with a protein depletion of 97% after SXC and > 99% after SCMA, resulting in protein concentrations of ~ 15 µg/mL after SXC and < 2 µg/mL after SCMA (Fig. 6A,C, Table 1). The DNA depletion was 86% after SXC and 98% after SCMA compared to the initial feed concentration before nuclease treatment and SXC (Fig. 6A,B,D Table 1). In the SCMA eluate, the DNA concentration was 3 ng/mL at viral RNA titres of 3.2E+07 IU/mL (Fig. 6B,D, Table 1).

## Discussion

The DSP developed in this study consisted of clarification, ultrafiltration, nuclease treatment, and SXC and SCMA steps. While filtration-based clarification and concentration are commonly used initial process steps for the production of viral vaccines, a major rationale for using SXC for the virus capture was the predominant dependency on the size of the target species^28^. This promised an independence with regard to the specific HCV genotype and the robust depletion of smaller impurities. SCMA was selected for virus polishing, based on the heparin affinity of HCV^31,34^. Additionally, both methods allowed to fully exploit the advantages of membrane adsorbers, such as a higher capacity and convective flow properties, compared to packed-bed resins^20^. Another benefit of the chosen methodology is the possibility to directly load the SXC eluent to the SCMA—if necessary, by an inline dilution. Importantly, the described DSP showed a similar performance for two major HCV genotypes (1a and 5a), potentially facilitating the development of vaccines targeting different HCV genotypes.

Filter-based HCV clarification resulted in virtually complete virus recovery. For ultrafiltration, we observed that recovery varied between 50 and 100% with mean recovery of approximately 70% (numbers are based on described and additional unpublished experiments). According to these data, recovery was neither depending on the genotype of the processed virus nor on the size of the hollow fibre. This variation is likely due to suboptimal process control. In the laboratory facilities where infectious virus was handled no ultrafiltration system was available, and thus optimal control of pressure and flow rate was not possible. As ultrafiltration resulted in virtually complete HCV recovery in several experiments and as high recoveries for other viruses have been described using this methodology^39,40^, we anticipate that consistently high HCV ultrafiltration recoveries could be achieved given the possibility for optimal process control.

For SXC, the initial application of published process conditions^26^ did not result in a successful virus retention. It was previously described, that the SXC performance is optimal at pH conditions near the pI^28^. For HCV, no characterization of the pI of the complete virus has been published so far. However, Prasetyo et al*.* reported a pI of 9.3 for HCV virus-like particles comprised of HCV Core (protein specific pI: 11.5) and envelope proteins (protein specific pI 8.1)^36^. Further, for genotype 1a, Viprbrc.org provided a theoretical pI of ~ 12, 7.4 and 7.8 for the structural proteins Core, E1 and E2, respectively, based on computation from GenBank sequences. Testing alkaline pH conditions, we defined a small operating window for an optimal SXC performance at pH 9 ± 0.5. It has not yet been described how pH alterations affect SXC of other virus particles, such as the Influenza A virus^26^ and the Baculovirus^27^, which have neutral to acidic pI and a > 90% recovery in SXC at pH 7.4. For HCV, the intense pressure increase observed during SXC at pH 11 suggested severe membrane fouling, resulting in a nearly complete virus retention, which hampers elution. This might be caused by the precipitation of proteins, medium components, or virus particles under these conditions. It is not surprising that an impurity removal during the SXC was independent of the applied pH, as the chosen PEG concentrations were too low to sterically exclude the DNA and protein molecules^27^. At these conditions, a protein retention does not occur, and the observed DNA retention may be due to DNA attachment to the virus or to DNA co-purified inside extracellular vesicles as previously described by Marichal-Gallardo *et al*^26^. The membrane capacity during SXC was slightly higher than previously reported for the Baculovirus^27^, which could be explained by the smaller size of HCV. The pressure increase during DBC determination may be due to the possible aggregation potential of the virus, leading to increased membrane fouling for higher loading volumes. It is not likely that an increased pore blockage is caused by protein impurities, as these are mostly washed out during sample application, with mainly virus particles remaining on the column. On a process scale, the pressure limitations might be additionally reduced as well as the binding capacity increased by using a different type of membrane housing, offering an altered angle of the incident flow.

Our data highlights the importance of avoiding a freeze–thaw cycle in an SXC elution buffer preceding SCMA, which resulted in a large decrease in recovery, possibly due to a degradation of HCV particles, or changes in the surface protein composition or structure. The decreased virus stability during freeze-thawing might be caused by removal of the proteins during SXC, which might stabilize the virus and prevent degradation. Although, in general, storage times using freeze–thaw cycles are unusual during a production process, this information may support similar trials in other laboratories. With regard to virus recovery, no significant differences (according to a students’ T-test, data not shown) were observed for samples that had been subjected to a nuclease treatment + SXC prior to SCMA (50% for 1a and 49% for 5a HCV) compared to samples that had been processed by SXC only prior to SCMA (63% for 1a HCV), with respect to the analytical error. Thus, SCMA appeared to be unaffected by a preceding nuclease treatment and independent of the virus genotype. However, SCMA recoveries were below the values previously described for the Influenza A virus^31^. Fortuna et al*.* reported 75–81% recovery for the Influenza A virus, using the same type of membranes. Furthermore, a low conductivity and a high virus titre of the virus feed were reported to be required for an optimal SCMA performance^32^. While we maintained the conductivity during sample application below 5 mS/cm, the virus was diluted 10 times after SXC in order to obtain these conditions. Thus, the SCMA recovery may be further increased by interposing a concentration and diafiltration step, reducing both the sample volume and the conductivity, which might also allow a reduction of the processing time. Furthermore, an optimization of the membrane ligand density as previously done for Influenza A virus-like particles could improve the virus retention and reduce losses during loading^33^.

We observed a highly efficient protein depletion. Within the analytical error, a virtually full protein depletion could be achieved by SXC, with a protein removal of > 99% for 1a HCV and 97% for 5a HCV. Using the Micro BCA assay, following SXC protein concentrations of 4 µg/mL (1a HCV) and 15 µg/mL (5a HCV) were determined with a further reduction to 0.9 µg/mL (1a HCV) and 1.5 µg/mL (5a HCV) following SCMA. These results were verified using silver staining of SDS-PAGE gels, also suggesting efficient protein depletion in SXC and removal of residual protein in SCMA (data not shown). The fact that protein could not be detected in the SCMA flow-through and wash fractions might be due to the sample dilution preceding the SCMA. Since the SCMA elution resulted in a sample concentration, the low concentration of proteins in the final HCV product (< 2 µg/mL) suggested a successful depletion of the remaining proteins during SCMA.

A comparable DNA depletion of at least 98% was achieved for both viruses of both HCV genotypes following a nuclease treatment, SXC, and SCMA. Of this overall DNA depletion, 5–12% were achieved by SCMA, whereas the nuclease treatment allowed an additional removal of about 10% of the total DNA. At present, it is not known, which amount of HCV particles a vaccine should contain to induce an efficient immune response. If required, an additional DNA reduction may be achieved by optimizing the nuclease treatment regarding the enzyme concentration and the incubation time. Most likely, the remaining DNA represents fragments attached to the virus as described above, or DNA being co-eluted with the virus particles using 0.6 M NaCl as SCMA elution buffer. For the latter, a further optimization of the SCMA procedure, including the evaluation of the virus elution using buffers with lower conductivity, is conceivable. Additionally, it should be mentioned, that the entire amount of DNA in the SCMA feed could not be recovered in the subsequent fractions. This might be caused by the DNA remaining on the column, and by an inhomogeneous error distribution between the varying salt concentrations.

We provide proof-of-concept for a novel DSP for purification of cell culture-derived HCV facilitating development of a whole virus vaccine. In future studies it will be important to demonstrate scalability of this DSP. Purification of larger amounts of HCV will facilitate more detailed studies of the potential vaccine antigen including analysis of virus structure by electron microscopy and quantification of HCV structural proteins.

Further, chromatographic experiments were carried out using inactivated HCV, as preferable for an industrial vaccine production. In future studies it would be of interest to process non-inactivated HCV to confirm that SXC and SCMA do not have a negative impact on HCV infectivity, implying a preservation of structural integrity. Importantly, it has previously been reported for other virus particles, that neither of the methods affected virus infectivity^26,27,31^.

We showed process robustness for two different HCV genotypes with significant sequence difference in the structural proteins Core, E1 and E2, which are incorporated in the viral particle. As all cell culture viable HCV recombinants, genotype 1a and 5a viruses used in this study contained cell culture adaptive substitutions. While for genotype 5a an early passage virus was used recapitulating the sequence of the most cell culture adapted clone available^41^, for genotype 1a a passage 20 virus was used showing a higher degree of genetic heterogeneity and differing at several amino acid positions compared to the less cell culture adapted original clone^35^. However, for the genotype 1a passage 20 virus most sequence heterogeneity was found in the nonstructural proteins, which are thought not to be incorporated in the HCV particle. The influence of cell culture adaptive substitutions in the structural proteins on immunogenicity remains to be determined in future studies.

Finally, in future studies it would be of interest to evaluate applicability of the developed DSP to the purification of HCV virus-like particles consisting of HCV structural proteins. During clarification different filter pore sizes and for SXC, an adjustment of the PEG concentration might be required in case of varying product sizes. The use of SCMA will require that virus-like particles maintain pseudo-affinity to sulfated cellulose.

For HCV vaccine development an inactivated whole virus approach is attractive, given the intricate conformation of the envelope proteins, which is difficult to mimic in subunit envelope vaccines. Further, given the higher immunogenicity of whole viruses compared to viral envelope proteins^17^, and the historic success of whole virus vaccines^16^, whole viruses are attractive vaccine antigens. This approach has only become feasible due to the relatively recent development of cell culture systems for the production of HCV^42^. However, further studies are needed to elucidate which HCV genotypes, possibly with certain envelope modifications for exposure of conserved epitopes, have the highest immunogenicity.

## Materials and methods

### Huh7.5 cell culture

Huh7.5 cells (obtained from Apath, LLC; New York, USA) were maintained in DMEM (Gibco) with 10% fetal bovine serum (Sigma) and penicillin (100 U/mL) / streptomycin (100 µg/mL) (Sigma) and were incubated at 37 °C and 5% CO_2_. Adenovirus Expression Medium (AEM) (Gibco), supplemented with penicillin (100 U/mL) and streptomycin (100 µg/mL), was used for HCV production under serum-free conditions^38^.

The percentage of HCV infected cells was evaluated by immunostainings^38,43^. In brief, cells were seeded in a chamber slide (Thermo Fisher Scientific) for a confluent cell layer, fixed with acetone (Merck) the next day, and stained with primary antibody 9E10 diluted 1:3,000^44^, followed by secondary antibody Alexa Flour 488 goat anti-mouse IgG diluted 1:500 (Invitrogen), and Hoechst 33342 (Molecular Probes) diluted 1:1,000.

HCV-infectivity titres were determined with three technical replicates as FFU/mL in a cell-based assay in 96-well plates as described^45,46^. The immunostaining of 96-well plates was carried out with primary antibody 9E10 diluted 1:5,000, secondary antibody ECL Anti-mouse IgG Horseradish Peroxidase linked from sheep (Amersham Biosciences) diluted 1:500, and visualized with Pierce DAB Substrate Kit (Thermo Scientific). 96-well plates were imaged and automatically counted for FFU quantification.

### Serial passage for generation of high-titre genotype 1a HCV

For the production of genotype 1a HCV, the cell culture infectious recombinant TNcc^35^ was further adapted to cell culture by serial passage in Huh7.5 cells. Following a transfection of HCV RNA transcripts, 18 viral passages to naïve cells were carried out in T80 cell culture flasks (Nunc). Naïve cells were inoculated with cell culture supernatant derived from the previous culture at the peak of infection according to immunostainings as described^41^. A passage 19 virus seed stock was prepared in T500 triple layer flasks (Nunc TripleFlask Treated Cell Culture Flask); supernatants from two time points at the peak of infection were pooled.

### Production of genotype 1a and 5a HCV for DSP development

For genotype 1a HCV, T175 flasks, seeded with 6 × 10^6^ cells in DMEM on the previous day, were inoculated at a multiplicity of infection of 0.003 with the passage 19 virus seed stock. Cultures were expanded to T500 triple layer flasks. When 80% of cells were estimated to be infected by immunostaining in a replicate T25 culture, the cultures were washed with PBS (Sigma) and subsequently maintained in AEM under serum-free conditions. The supernatant was harvested five times every 2–3 days, yielding 10.5 L, which was stored at − 80 °C until further processing.

For genotype 5a HCV, 18 × 10^6^ cells, seeded the previous day in DMEM, were infected at a multiplicity of infection of 0.003 in T500 triple layer cell culture flasks with a 3rd passage seed stock of the further adapted SA13/JFH1 recombinant^37,41^. The following day, cells were transferred to cell factories (Nunc Cell Factory). When 80% of cells were expected to be infected as indicated by immunostaining in a replicate T25 culture, the cells were washed with PBS and cultured in AEM. The harvesting of the supernatant was carried out five times every 2–3 days, yielding 20.4 L total, stored as described above.

### Sequence analysis

NGS of the virus populations was carried out as described^47,48^. Briefly, RNA was extracted with Trizol LS and the RNA Clean & Concentrator-5 (Zymo research) kit. The reverse transcription was carried out with Maxima H Minus Reverse Transcriptase (ThermoScientific), the whole open reading frame was amplified with polymerase chain reaction (PCR) Q5 Hot start High-Fidelity DNA Polymerase (New England Biolabs), and the PCR product was purified (DNA Clean & Concentrator-25 and Zymoclean Large Fragment DNA Recovery Kit, Zymo research). The NEBNext ultra II FS DNA Library Prep Kit (New England Biolabs) was used for library preparation, and sequencing was performed with an Illumina Miseq platform.

The alignment of amino acid sequences of structural proteins (Core, envelope proteins E1 and E2) of 1a and 5a HCV was done in BLAST (database version 5; https://blast.ncbi.nlm.nih.gov/Blast.cgi)^49,50^.

### Evaluation of infectious HCV stability at alkaline pH values

The HCV genotype 5a seed stock described above, was concentrated using Ultra-15 Centrifugal Filter Unit-100 K (Amicon) and diluted by a factor 17 in PBS (for pH 7.4 reference), DMEM (standard cell culture medium, pH 8.5) or phosphate buffer (KH_2_PO_4_ (Sigma) and K_2_HPO_4_ (Sigma), adjusted with NaOH for pH values of 9.5, 10 and 11) in triplicate Eppendorf tubes, and incubated for 90 min at room temperature. After incubation, the virus/buffer solutions were diluted 1:40 in DMEM containing 20 mM HEPES (HEPES solution 1 M, Sigma), and added to triplicate wells seeded with 7 × 10^3^ cells/well in 96-well poly-D lysine plates (Thermo Scientific) the previous day. The infected cell plates were incubated for six hours at 37 °C and 5% CO_2_ before the medium was exchanged to DMEM without HEPES. The cell plates were fixed, stained, and evaluated as described above, in order to quantify HCV infected single cells. The cell viability was evaluated after the experimental read-out had been obtained in a replicate experiment with the CellTiter 96 AQueous One Solution Cell Proliferation Assay (Promega) according to the manufacturer’s protocol. The pH effect on virus stability was evaluated as the number of HCV-positive cells in a well, relative to the average number of HCV-positive cells obtained from the virus incubated at a pH value of 7.4.

### Virus clarification, ultrafiltration and inactivation

The genotype 1a and 5a virus material was passed over 5 µm and 0.65 µm Sartopure PP3 (Sartorius) capsule filters for a two-step clarification by peristaltic pumping (Masterflex L/S 7554–95 Cole-Parmer, Masterflex L/S Easy Load pump head, and Extended Lifetime Silicone Tubing size 17 (Repligen)). Subsequently, the clarified 1a and 5a viruses were concentrated 249 and 325 times, respectively, in two sequential ultrafiltration steps with hollow fibre filters (MINIKROS SAMPLER 65CM 500 K MPES 0.5MM 3/4TC X 3/4TC STERILE followed by MINIKROS SAMPLER 20CM 500 K MPES 0.5MM 3/4TC X 3/4TC STERILE, both Repligen). The HCV infectivity titres and RNA titres were determined for samples from each step of clarification and concentration. The virus recovery was calculated from the HCV RNA titres.

The virus was inactivated by UV exposure (UVP Handheld UV lamp, UVG-54 254 nm in lamp stand) in 6-well plates (Nunc) with 1.5–2.5 mL per well for eight hours. The 6-well plate was kept on ice with frequent agitation. To confirm inactivation, naïve Huh7.5 cells were seeded in triplicate T25 flasks (1 × 10^6^ cells/flask) the previous day, and inoculated with 20 µL of UV-treated material. Inoculated cultures were passaged for 21 days and monitored for HCV positive cells by immunostaining as described above. In replicate samples, it was confirmed that a similar incubation without UV irradiation did not inactivate HCV.

### Nuclease treatment

The nuclease treatment was performed in triplicates for both genotypes. The clarified virus was subjected to 250 U/mL Benzonase nuclease (Merck) at a final concentration of 2 mM MgCl_2_. The incubation was done overnight at 4 °C, and the nuclease activity was blocked afterwards, using a final concentration of 5 mM EDTA. Subsequently, the chromatography was performed, using nuclease–digested, clarified HCV.

### Chromatographic purification

The chromatographic experiments were done with an Äkta Pure 25 system, operated by Unicorn (version 7.1, GE Healthcare Life Sciences). Online monitoring was done by system-integrated UV (280 nm) and conductivity detectors, and additionally light-scattering was detected with a Nano DLS Particle Size Analyzer (Brookhaven Instruments). All chromatographic experiments were done in technical triplicates, unless stated otherwise.

### Virus capture using SXC

SXC was performed using regenerated cellulose membranes with 1 µm pore size (Whatman), as previously reported^26,27^. In brief, for preparing the column, 10 membranes were punched and stacked into a 13 mm filter holder (Pall), yielding a total membrane area of 13.3 cm^2^. All steps were performed at a flow rate of 2 mL/min. The stack was equilibrated using 5–10 mL of 20 mM Tris at the specified pH value, supplemented with 180 mM NaCl, and 8% PEG 6,000. Clarified, concentrated, and inactivated HCV was mixed 1:4 with the above stated buffer and supplemented with 32% PEG to yield final concentrations of 8% PEG to match the equilibration conditions. After sample application, the stack was washed with equilibration buffer until the detector signals decreased to baseline (> 5 mL). Elution was achieved using 20 mM Tris at pH 7.4 without PEG, but supplemented with 0.4 M NaCl. Initial screening SXC runs were tested at pH 7.4 to pH 11 for genotype 1a HCV, while final process conditions were at pH 9, and tested for robustness at pH 8.5 and 9.5. Following optimization, the SXC performance was verified for the genotype 5a HCV at pH 9 with a preceding nuclease treatment.

### Virus polishing using SCMA

Sartobind Sulphated Cellulose membranes with a nominal pore size of 0.8 µm (Sartorius Stedim Biotech GmbH) were punched to disks of 13 mm diameter. As for SXC, the disks were stacked to layers of 10 membranes (13.3 cm^2^ total membrane area). All steps were performed at a flow rate of 0.8 mL/min. Membranes were equilibrated using 20 mM Tris pH 7.4 prior to sample application. For the purification, SXC elution fractions were diluted 1:10 with equilibration buffer in order to reduce the conductivity of the solution below 5 mS/cm. After complete sample loading, the membranes were washed with equilibration buffer until UV- and light scattering signals returned to baseline. Bound components were subsequently eluted in 3 fractions, using increasing NaCl concentrations (0.6, 1.2 and 2 M). The SCMA was evaluated for genotype 1a HCV, using SXC elutions with and without an interim storage at − 80 °C, as well as with and without additional nuclease treatment before SXC. Finally, the SCMA performance was confirmed, using a nuclease-treated and SXC-purified genotype 5a HCV.

### Determination of dynamic binding capacities

For SXC and SCMA, the DBC was determined in order to optimize the virus load on the membrane stacks. Stationary and mobile phase compositions were the same as described above. A clarified, concentrated, and inactivated virus feed of a known concentration was prepared and applied to the column, until detected breakthrough of 10% and 100% of the particles, based on the evaluation of the light-scattering detector signal. Depending on the loaded volume, the total amount of virus particles, at which breakthrough rates of 10% or 100% (DBC_10_ and DBC_100_) occurred, was calculated and related to the area of the membrane. All process runs were performed at or below DBC_10_.

### HCV quantification

The virus amount was evaluated using an in-house qPCR as described previously, with minor modifications^43^. Briefly, viral RNA was extracted from 200 µL sample and eluted in 50 µL water, using the High Pure Viral Nucleic Acid Kit (Roche) according to the manufacturer´s instructions. Afterwards, a mixture comprising TaqMan Fast Virus 1-step Mastermix (Thermo Fisher Scientific), nuclease-free water, probe (containing a FAM dye and an MGB quencher) and primers (Sigma-Aldrich) was prepared. 12 µL of that mixture were added to 8 µL of the extracted RNA in a 96-well PCR plate (twin.tec, Eppendorf) preparing duplicates for each sample. The amplification was done using a Mastercycler Ep gradient S realplex (Eppendorf) after a pre-incubation period at 50 °C for 300 s. A total of 53 cycles of 95 °C for 20 s, followed by 62 °C for 60 s, were performed. An HCV standard panel, containing 10^2^ to 10^6^ IU/mL in 1-log increments, was prepared and included in each run, in addition to negative control samples. HCV RNA titres (IU/mL) were calculated using a standard curve generated from values obtained for the standard panel and corresponding cycle threshold values. The standard deviation of triplicate measurements was below 20%.

### Protein determination

For a quantification of the total protein amount contained in the chromatographic samples, the Pierce BCA Protein Assay Kit (Thermo Fisher Scientific) was applied according to the manufacturer’s instructions. In brief, 25 µL of sample were transferred into a clear 96-well plate; duplicates were prepared for each sample. The standard panel (in the range of 25 to 2,000 µg/mL) was prepared from gamma globulin according to the manufacturer’s instructions. To each well, 200 µL of the reaction mix were added, and absorbance at 562 nm was measured after 30 min of incubation at 37 °C using the Cytation 3 plate reader (BioTek). Additionally, product fractions of the two purification steps (SXC and SCMA elutions) were analysed using the Pierce Micro BCA Protein Assay Kit, offering a lower calibration range between 0.5 and 200 µg/mL. Sample preparation was done as instructed by the manufacturer in a 96-well format and measurements were obtained using the same equipment and absorbance as described above. For both approaches, the values obtained from a blank sample (buffer) were subtracted before interpolating the sample concentrations. Results given are from duplicate measurements with less than 10% standard deviation.

### DNA determination

The total amount of double stranded DNA (referred to as “DNA” in this work) was determined, using the Quant-iT PicoGreen dsDNA Kit according to the manufacturers´ instructions. The assay was performed in a 96-well format, using black microtiter plates (Nunc). Chromatographic samples, including the feed, were mixed 1:4 (SXC samples) or 1:2 (SCMA samples) with the assay’s 1 × TE buffer to a final volume of 100 µL. For each plate, blank samples (buffer) and two standard panels were prepared from kit-contained lambda-DNA in the range of 1 to 1,000 ng/mL and 0.025–25 ng/mL, using a tenfold dilution series. After adding 100 µL of the reaction dye, the plate was incubated for 5 min in the dark, and a fluorescence emission at 520 nm (excitation: 485 nm) was subsequently determined, using the Cytation 3 plate reader (BioTek). All measurements were done in duplicates with a general standard deviation of less than 10%.

## Supplementary information

Supplementary Information.

## Abbreviations

AEM: Adenovirus expression medium
DAA: Direct-acting antivirals
DBC: Dynamic binding capacity
DMEM: Dulbecco’s modified eagle medium
DSP: Downstream process
FFU: Focus forming units
GMP: Good manufacturing practices
HCV: Hepatitis C virus
Huh7.5: Human hepatoma cell line 7.5
IU: International units
NGS: Next generation sequencing
PCR: Polymerase chain reaction
pI: Isoelectric point
PBS: Phosphate buffered saline
PEG: Polyethylene glycol
qPCR: Quantitative polymerase chain reaction
SCMA: Sulphated cellulose membrane adsorber
SD: Standard deviation
SXC: Steric exclusion chromatography
