# Supplementary material for: Development of a downstream process for the production of an inactivated whole hepatitis C virus vaccine
Source: Sci Rep. 2020 Oct 1;10:16261. doi: 10.1038/s41598-020-72328-5 (PMC7530675; doi:10.1038/s41598-020-72328-5)
Supplement: Supplementary file 1 — Supplementary Information. [file 41598_2020_72328_MOESM1_ESM.pdf]

Development of a downstream process for the production of an inactivated whole hepatitis C virus vaccine

Keven Lothert[1*]; Anna F. Offersgaard[2*]; Anne F. Pihl[2]; Christian K. Mathiesen[2], Tanja B. Jensen[2]; Garazi Peña Alzua[2], Ulrik Fahnøe[2]; Jens Bukh[2]; Judith M. Gottwein[2$]; Michael W. Wolff[1,3$]

1. Institute of Bioprocess Engineering and Pharmaceutical Technology, Technische Hochschule Mittelhessen – University of Applied Sciences, Giessen, Germany

2. Copenhagen Hepatitis C Program (CO-HEP), Department of Infectious Diseases, Hvidovre Hospital and Department of Immunology and Microbiology, Faculty of Health and Medical Sciences, University of Copenhagen, Copenhagen, Denmark

3. Fraunhofer Institute for Molecular Biology and Applied Ecology (IME), Giessen, Germany

*, Authors contributed equally

$, Authors contributed equally

Corresponding authors: Judith M. Gottwein (jgottwein@sund.ku.dk),
Department of Infectious Diseases,
Copenhagen University Hospital, Hvidovre
Kettegård Alle 30, 2650 Hvidovre, Denmark

and

Michael W. Wolff (michael.wolff@lse.thm.de)
Department of Life Science Engineering
University of Applied Sciences Mittelhessen
Wiesenstrasse 14, 35390 Giessen, Germany

Supplementary Results

Protein	Core	E2	E2	NS2	NS2	NS2	NS2	NS4B	NS4B	NS5A	NS5A
Nucleotide position	373	1571	2464	2822	2824	2935	3364	5812	6067	6729	7296
Original nucleotide	C	C	G	G	C	A	G	G	G	C	C
Acquired nucleotide	A	G	A	A	T	C	A	A	C	A	T
Allele frequency (%)	44	46	22	29	21	26	48	27	75	45	28
Amino acid change	T11N	N410K	S708N	M827I	A828V	N865T	R1008Q	G1824D	G1909A	L2130I	P2319S

Protein	…	NS5A	NS5A	NS5A	NS5A	NS5A	NS5B	NS5B	NS5B	NS5B	NS5B
Nucleotide position	…	7464	7522	7588	7591	7596	7785	8292	8985	9045	9298
Original nucleotide	…	A	A	A	T	T	A	A	A	A	A
Acquired nucleotide	…	G	G	G	C	C	G	C	C	C	G
Allele frequency (%)	…	23	21	26	49	23	46	83	28	47	98
Amino acid change	…	S2375G	E2394G	D2416G	V2417A	C2419R	S2482G	N2651H	I2882L	I2902L	H2986R

Supplementary Table S1: Next generation sequencing analysis of the open reading frame of the 1a HCV virus production. The sequence of 1a virus produced for DSP development (passage 20) was analyzed. Nucleotide and protein positions are stated relative to the TNcc sequence (GenBank accession no. JX993348) and are equivalent to positions in the 1a H77 sequence (GenBank accession no. AF009606). Positions with changes of at least 20% population prevalence are included in the table.
